# Expanding the Species and Chemical Diversity of *Penicillium* Section *Cinnamopurpurea*

**DOI:** 10.1371/journal.pone.0121987

**Published:** 2015-04-08

**Authors:** Stephen W. Peterson, Željko Jurjević, Jens C. Frisvad

**Affiliations:** 1 Bacterial Foodborne Pathogens and Mycology Research Unit, National Center for Agricultural Utilization Research, Agricultural Research Service, U.S. Department of Agriculture, 1815 North University Street, Peoria, Illinois, 61604, United States of America; 2 EMSL Analytical, Inc., 200 Route 130 North, Cinnaminson, NJ, 08077, United States of America; 3 Technical University of Denmark, Søltofts Plads, Bldg. 221—DTU Systems Biology, Kgs. Lyngby DK-2800, Denmark; University of Wisconsin—Madison, UNITED STATES

## Abstract

A set of isolates very similar to or potentially conspecific with an unidentified *Penicillium* isolate NRRL 735, was assembled using a BLAST search of ITS similarity among described (GenBank) and undescribed *Penicillium* isolates in our laboratories. DNA was amplified from six loci of the assembled isolates and sequenced. Two species in section *Cinnamopurpurea* are self-compatible sexual species, but the asexual species had polymorphic loci suggestive of sexual reproduction and variation in conidium size suggestive of ploidy level differences typical of heterothallism. Accordingly we use genealogical concordance analysis, a technique valid only in heterothallic organisms, for putatively asexual species. Seven new species were revealed in the analysis and are described here. Extrolite analysis showed that two of the new species, *P*. *colei* and *P*. *monsserratidens* produce the mycotoxin citreoviridin that has demonstrated pharmacological activity against human lung tumors. These isolates could provide leads in pharmaceutical research.

## Introduction

*Penicillium* is a mold genus widely known for production of the antibiotic penicillin by some species, ripening of camembert and blue cheeses by others, and the production of damaging mycotoxins in feeds, forage and foods by yet other species [1–3]. Monographic treatments of *Penicillium* based on morphology and physiology [4] resulted in the splitting of *Penicillium* into four subgenera, and subsequent phylogenetic study justified moving subgenus *Biverticillium* species into *Talaromyces* [5, 6]. The genus is also a source for compounds that possess therapeutic activities [7].

Nomenclatural rules of the past have allowed dual naming where the sexual and asexual morphs of a single species were placed in different genera. A new nomenclature [8] rescinds the prior rule and requires a single name for a single species. We follow the examples of others [9, 10, 11] in using the name *Penicillium* to include species with a *Eupenicillium* sexual state. There is general agreement to this choice in the mycology community [12].

Citreoviridin is a neurotoxic mycotoxin first isolated from yellow rice and is believed to be the cause of acute cardiac beriberi disease in humans [3]. Citreoviridin contaminated commodities are uncommon but acute cardiac beriberi was recently reported in Brazil [13, 14] and associated with moldy rice. Citreoviridin has also been reported as a contaminant of maize [15–16] and pecans [17] in the USA and frozen processed chicken in Brazil [18].

Morphological definition and recognition of *Penicillium* species has been contentious with one authority accepting either many more or many less species than another [1, 4]. Biological species concepts (BSC) introduced the notion of gene pools and gene flow as a defining characters of species [19] but its implementation in fungi was hindered because many species are either exclusively asexual or have homothallic mating systems. A system for establishing species boundaries based on multi-locus DNA sequence concordance analysis was introduced for fungi [20–22], but like the BSC, concordance analysis is only appropriate in species with heterothallic mating systems. The phylogenetic species concept [23] recognizes independently evolving lineages but defining species boundaries in clonally propagating lineages is an unresolved issue. Barcode identification of fungi was proposed to use genotypic data [24, 25] no matter how we define fungal species.

During a study of *Penicillium* species parasitizing *Aspergillus* conidial heads [26, 27] two new *Penicillium* species were described but one isolate in that study (NRRL 735) was left as an unnamed species because some authorities question the morphological stability of NRRL 735 [1, 4]. In this study we used barcoding theory [24, 25] to detect and assemble a set of isolates similar or identical to NRRL 735 in order to describe this new species with additional isolates. The assembled isolates were sequenced at six loci for phylogenetic analysis and grown on several media for morphological analysis. Extrolite profiles were determined because one isolate included in the study is known to produce the mycotoxin citreoviridin [17]. Seven new species were identified and described with extrolite data, a table of species known to produce citreoviridin is provided and DNA sequence data from this study were placed in a publicly accessible database for species identification [28].

## Materials and Methods

### Cultures

The cultures used in this study are available from the Agricultural Research Service Culture Collection (NRRL) Peoria, IL [29] and are also deposited at the IBT culture collection, Technical University of Denmark, Lyngby, Denmark. Working stocks of the cultures were maintained on potato dextrose agar slants for the duration of the study. Provenance of the isolates is detailed in S1 Table.

### Isolation techniques

Many of the isolates used in this study originated from indoor air samples. The air sampler design and use was detailed previously [30] and the isolation medium was malt extract agar (MEA). Dilution plates were used to isolate fungi from soil [31].

### Morphological analysis

For phenotypic evaluation, cultures were grown for 7 and 14 d on CYA and MEA agars [4]. Additional growth media were Difco Potato Dextrose agar (PDA), oatmeal agar (OA) [32], Czapek’s yeast autolysate agar with 20% sucrose (CY20S) and Czapek’s yeast autolysate agar supplemented with 5% NaCl (CYAS). Specific color names are from the Ridgway color guide [33] and are indicated by a parenthetical R and plate number. Microscopy, microphotography and macrophotography were as described [30] using acid fuchsin dye. Photographs were resized and modified for contrast using Adobe Photoshop Elements 10 [34].

### DNA extraction sequencing and analysis

Biomass for DNA extraction was grown, DNA was isolated and purified, and loci were amplified and sequenced as described [30]. Sequenced loci were beta tubulin (*BT2)*, calmodulin (*CF)*, nuclear internal transcribed spacer (*ITS)*, minichromosome maintenance factor 7 (*Mcm7)*, DNA dependent RNA polymerase II subunit (*RPB2)*, and ribosome biogenesis protein (*Tsr1)*. Sequences were deposited in GenBank and the accession numbers are listed (S2 Table). An instance of BIGSdb [35] was implemented and populated with sequences from this study and associated GenBank sequences for public fungal identification searches [28].

Maximum likelihood analysis was performed using Mega ver. 6.06 [36]. Sequences were aligned using Muscle, most appropriate model was tested, maximum likelihood was calculated and 1000 bootstrap replicates were conducted using the built-in functions of Mega. Aligned data files in Mega format are accessible as supporting information (S1–S6 Datafiles). Tree diagrams were opened in and annotated using CorelDraw X6 [37].

### Extrolite detection

Citreoviridin and other metabolites were induced and detected using described methods [38].

### Nomenclature

The electronic version of this article in Portable Document Format (PDF) in a work with an ISSN or ISBN will represent a published work according to the International Code of Nomenclature for algae, fungi, and plants, and hence the new names contained in the electronic publication of a *PLOS ONE* article are effectively published under that Code from the electronic edition alone, so there is no longer any need to provide printed copies.

In addition, new names contained in this work have been submitted to MycoBank from where they will be made available to the Global Names Index. The unique MycoBank number can be resolved and the associated information viewed through any standard web browser by appending the MycoBank number contained in this publication to the prefix http://www.mycobank.org/MycoTaxo.aspx?Link=T&Rec=. The online version of this work is archived and available from the following digital repositories: PubMed Central, LOCKSS.

## Results

**Fig 1:**
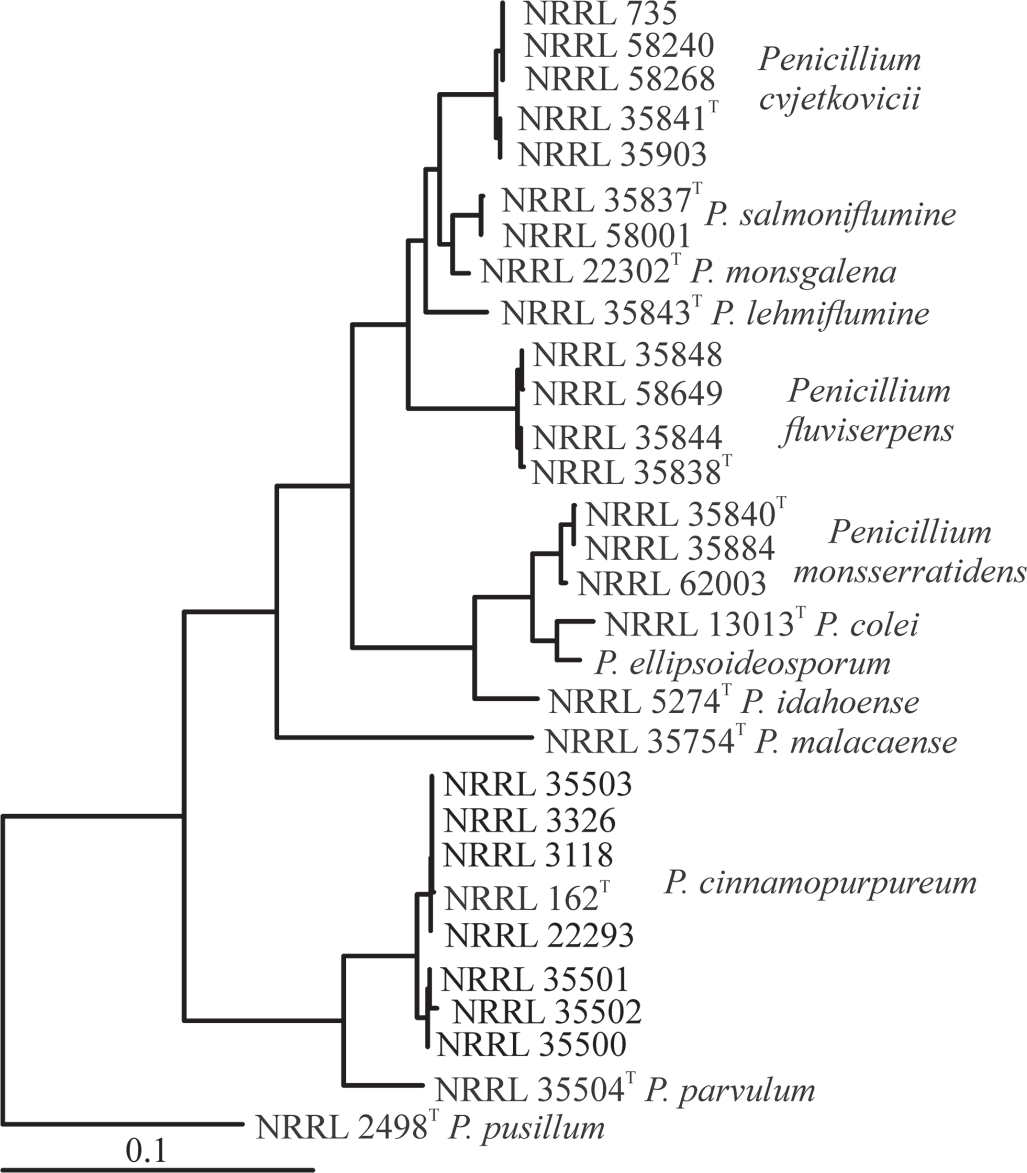
Phylogenetic tree of section *Cinnamopurpurea*. Fully resolved phylogenetic tree based on combined sequence data from *BT2*, *CF*, *Mcm7*, *RPB2* and *Tsr1*, computed with maximum likelihood in Mega. In the bootstrap, all nodes were above 95%, except the *P*. *fluviserpens* subclade NRRL 35844, NRRL 58649 77%; *P*. *cinnamopurpureum* subclade NRRL 35501-NRRL 35502 55%; and *P*. *cinnamopurpureum* subclade NRRL 162-NRRL 3118-NRRL 3326-NRRL 35503 64%). Outgroup chosen based on prior work [27]. *P*. *ellipsoideosporum* is represented only by sequences obtained from GenBank.

The datasets examined contained, by locus, *BT2*, 489 aligned coding and non-coding characters, best model K2+G; *CF*, 741 aligned coding and non-coding characters, best model K2+G; *ITS*, 533 aligned characters from ITS1, ITS2 and 5.8S rDNA, best model JC+I; *Mcm7*, 616 aligned coding characters, best model K2+I; *RPB2*, 1014 aligned coding characters best model K2+G; and *Tsr1*, 816 coding characters, best model T92+G. In each of the single locus trees (S1 Fig.) isolates formed terminal groups with strong statistical support. *Penicillium cvjetkovicii*, *P*. *lemhiflumine* and *P*. *fluviserpens* are strongly supported at 6 of 6 loci, *P*. *monsgalena*, *P*. *monsserratidens* and *P*. *idahoense* are supported by 5 of the 6 loci, and *P*. *colei* and *P*. *salmoniflumine* are supported by 4 of the 6 loci. Some species and terminal groups had very low bootstrap support at the ITS locus, but the ITS region is an effective barcode for these species, with the five protein coding loci also useful for genotypic species recognition. *Mcm7* and *RPB2* loci display the lowest intraspecific variation and would be most suitable for diagnostic purposes. The datasets were concatenated and analyzed providing a fully resolved view of the group (Fig. 1).

**Fig 2:**
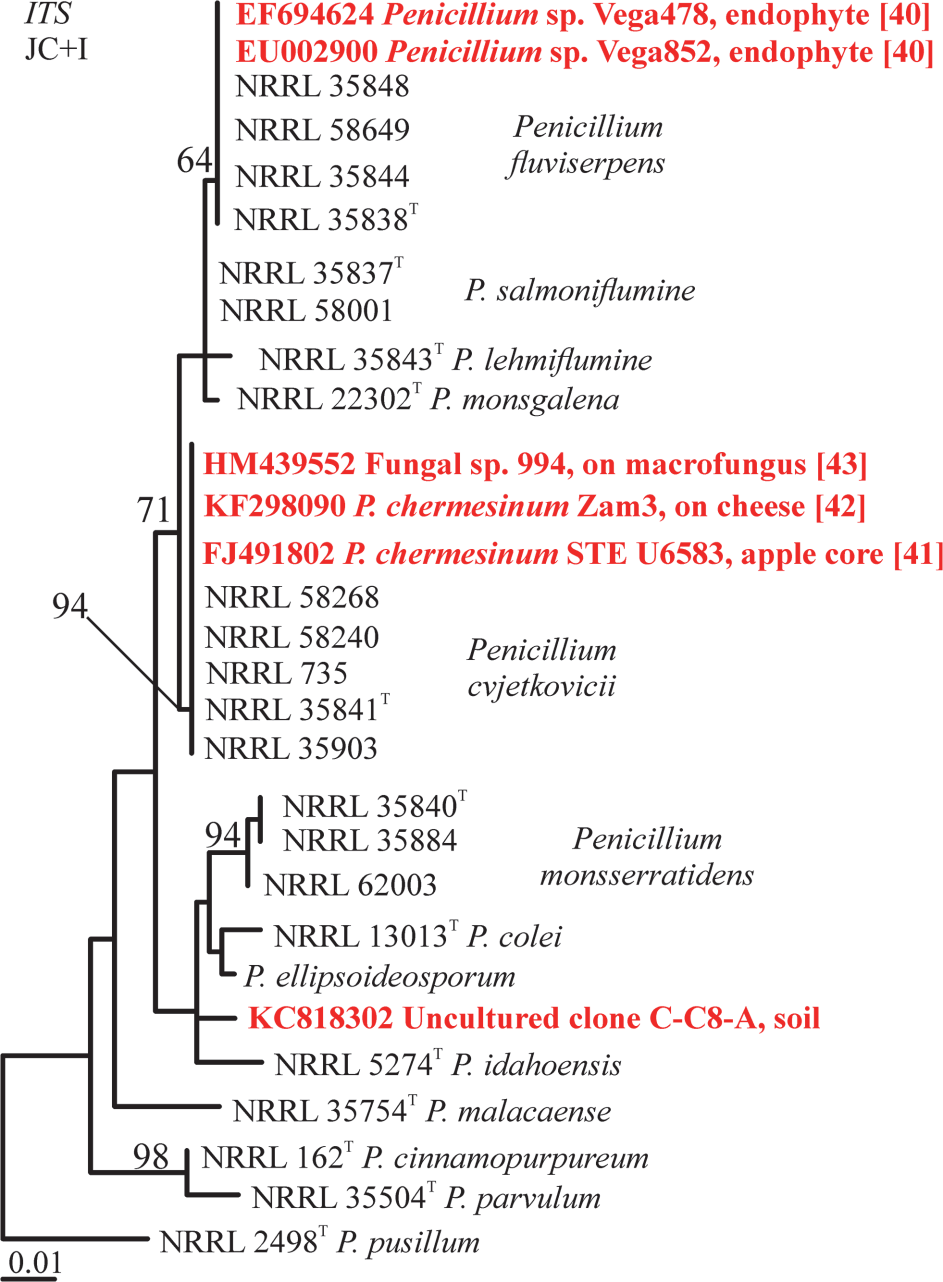
Phylogenetic tree based on ITS region sequences, with very similar sequences from GenBank. Unidentified endophytes from coffee plants in South America are *P*. *fluviserpens*; a cheese isolate from Europe, an apple core isolate from South Africa, and a fungal epiphyte from China are identified as *P*. *cvjetkovicii* although two were initially identified as *P*. *chermesinum* using phenotypic identification; and one sequence amplified from soil DNA represents an apparent unknown species. Bootstrap values above 70% are placed on the branches.

GenBank databases were searched prior to writing (accessed 12 July 2014) for additional isolates having sequences fitting with the newly described species. While all six loci were used for BLAST searches, the ITS region returned the most sequences and the most diverse sequences. Those sequences were downloaded, aligned with the study-group sequences and a tree was generated (Fig. 2; conditions as for ITS data above). Two unidentified endophytes from coffee plants in Colombia [39] were identified as *P*. *fluviserpens*. An isolate from an apple in South Africa [40], a cheese contaminant from Spain [41], both previously identified as *P*. *chermesinum*, and an unidentified decay mold of *Ophiocordyceps sinensis* in China [42] were all identified as *P*. *cvjetkovicii*. A sequence derived from an uncultured DNA clone from forest soil in Switzerland GB# KC818302 appears to represent an undescribed species from *Penicillium* section *Cinnamopurpurea*.

**Fig 3:**
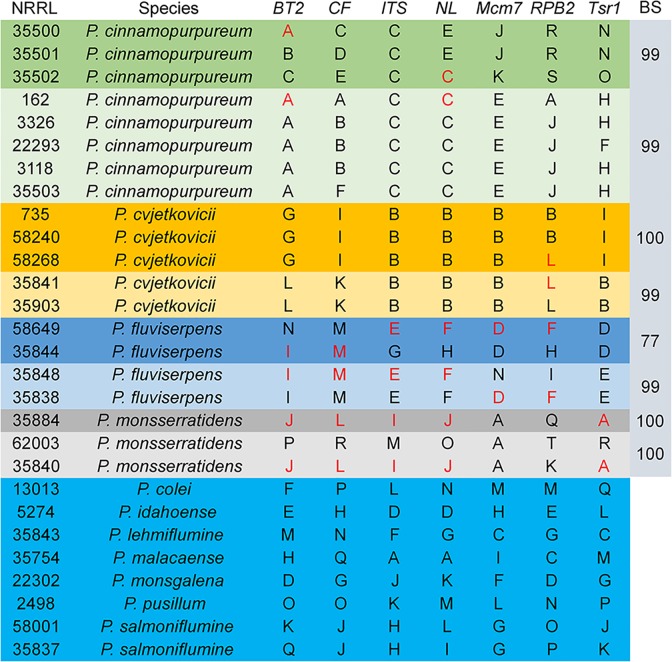
Haplotype analysis among isolates of section *Cinnamopurpurea* at seven loci grouped by high bootstrap values listed in the last column. At each locus unique haplotypes are given arbitrary letter designations. Subgroups with strong statistical support (Fig. 1) are shaded in a distinct color for the multi-isolate species. Haplotypes shared between distinct clades of a species are presented with red lettering.

The haplotypes of each isolate at each locus were compared and arbitrarily assigned letter designations (Fig. 3). In the figure, shading is used to represent groups of isolates with >90% bootstrap support in the multilocus tree (Fig. 1). Haplotypes are shared at some loci between subclades of species, but there are no shared haplotypes between species. The homothallic sexually reproducing species *P*. *cinnamopurpureum* has a pattern of haplotypes among the isolates similar to the patterns of the putatively clonal species *P*. *cvjetkovicii* and *P*. *fluviserpens*. Sharing haplotypes between strongly supported groups could result from incomplete lineage sorting [43] or from hyphal fusion of different genotypes in a homothallic species. Other species have only one or two representatives and patterns cannot be discerned.

Phenotypic data showed that these species grow slowly, rarely attaining a colony diameter exceeding 30 mm in 14d. Colony reverse colors and soluble pigments are consistent within species on particular media but as can be seen from the figures, the expression of these colored pigments is strongly influence by the growth medium. Other factors may influence expression because the colors do not always develop in repeated cultures of an isolate. The species produce mostly small (2–4 μm) conidia that vary from ellipsoidal to spherical with surfaces from smooth to slightly rough and a small number of much larger conidia. Conidiophores are furcate in this clade but *P*. *salmoniflumine* and *P*. *monsgalena* produce mainly unbranched penicilli. The presence of exudates in the species is variable with the medium and also can be variable among isolates of a single species. Character suites appear sufficiently stable and distinct for morphological diagnostic of the species. Diagnoses are listed with the descriptions of the species in the Taxonomy section.

Detailed extrolite data are listed with each isolate in the Taxonomy section. The in-group clade containing all the new species and *P*. *idahoense* have producers of the extrolites called OTO1–OTO6. These metabolites have UV spectra with absorptions at 218 nm (100%), 250 nm (35%) and 324 nm (23%), and retention indices (RI) at 746, 961, 824, 818, 748 and 846, respectively. Isolates in the sub-clade containing *P*. *colei* and *P*. *monsserratidens* produce citreoviridin and citreomontanin, whereas the sister group to these two, *P*. *idahoense* does not produce citreoviridins. One outgroup isolate *P*. *malacaense* NRRL 35754 does not produce any of the metabolites produced in the main clade. Several species in the main clade produce the metabolites named PR-828 (RI 828) and PR-878 (RI 878). The UV spectra of these extrolites resemble PR-toxin, sporogen AO1 and petasol, the latter two compounds earlier found in *Penicillium* sp. JP3430 [44]. These compounds have UV absorption at 251 nm, but mass spectrometric analysis is needed to identify these unknowns. The anthraquinone AQ-840 (RI 840) has approximately the same UV spectrum as carviolin (= roseopurpurin). Carviolin has been found in *P*. *roseopurpureum* and *P*. *dravuni* previously [45, 46]. The extrolite alk-752 (RI 752) had an indole chromophore and was only found in *P*. *lemhiflumine* and *P*. *fluviserpens*. The profiles of extrolites found in the new species and *P*. *idahoense* describe a closely related group of species that are also similar phenotypically.

The metabolite *AQ*-840 is common to all of the in-group isolates. Other metabolites such as *alk-752* may be produced by species of disparate clades (e.g., *P*. *fluviserpens* and *P*. *lemhiflumine*) or by a clade of species (e.g., metabolite *OTO1* present in the clade composed of *P*. *salmoniflumine*, *P*. *monsgalena and P*. *lemhiflumine*). Citreoviridin is produced in two of the new species, *P*. *colei* and *P*. *monsserratidens* that along with *P*. *ellipsoideosporum* form a clade in all of the single locus trees (S1 Fig.). Extrolite expression or detection in the isolates has little intra-specific variability (e.g., *P*. *fluviserpens* and *P*. *cvjetkovicii*, Taxonomy section).

## Discussion

Maiden and associates [47] introduced the technique of multilocus sequence typing (MLST) to bacterial systematics. A general theory of how to interpret the multilocus data for the definition of bacterial species is so far lacking. Practical frameworks for species limit designations are based on the amount of sequence difference observed in either typological species or species defined by the 70% DNA-DNA hybridization technique [48]. We provide an identification web site to go along with our phylogenetic taxonomy [28]. Genealogical concordance analysis [20–22] is effective in determining the limits of sexually reproducing species. Several factors were considered before applying this technique to this group of primarily anamorphic fungal species. All of the species in this clade are descended from the most recent common ancestor of *P*. *cinnamopurpureum* a species known to produce sexually derived ascospores and the in-group species. One of the in-group species, *P*. *idahoense*, makes meiotically produced ascospores. *Penicillium colei*, *P*. *cvjetkovicii*, *P*. *idahoense*, *P*. *lemhiflumine*, *P*. *monsgalena*, *P*. *monsserratidens* and *P*. *salmoniflumine* all produce a certain proportion of conidia that appear to be twice the size of the commonly observed small conidia. Conidium size has often been associated with ploidy level in fungi [49–52] and the distinct conidium sizes seen in these species suggests hyphal fusion and ploidy changes, all consistent with processes of heterothallic meiotic recombination. The polymorphisms at the different loci for the in-group are consistent with the polymorphism pattern of the out-group teleomorphic *P*. *cinnamopurpureum*. It is also notable that a number of *Aspergillus* and *Penicillium* species long thought to be strictly anamorphic have been shown in recent years to possess cryptic sexual states [53–57]. We hypothesize that our in-group species may also have cryptic sexual states.

Interspecific DNA sequence variation between sibling species is present at each locus along with limited intraspecific variation at some, and accurate species recognition by genotype relies on a database of existing species and accurate DNA sequencing of the unknown. The ITS region for barcoding fungi [24] can recognize all species in this group but some species differ by a single base at the ITS locus making absolute accuracy in sequencing essential. The other loci all exhibit larger interspecific differences and are more tolerant of varying sequencing accuracy. Some investigators have used sequences from three loci in their studies, most often ITS, beta-tubulin and calmodulin (e.g., [58, 59]) are chosen. The currently known species could confidently be identified using these three loci in an MLST scheme. The additional loci used in this study are essential for defining the genetic limits of the species using genealogical concordance analysis.

Phenotypic recognition of *Penicillium* species is complicated by a number of seemingly minor variations in culture conditions and isolate handling that can have large effects on the development of the characters used in phenotypic recognition of the species [60]. Aeration, depth of media in petri plates, size of inoculum, source of the yeast extract and other conditions can all have major impact on the phenotype of a culture [61, 62]. Efforts has been made to standardize growth conditions [63]. Certain aspects of culture handling such as initial single spore initiation of cultures [64] are essential for all methods of species identification.

Morphologically the species in section *Cinnamopurpurea* are quite similar, all producing subglobose to ellipsoidal smooth to finely roughened spores, monoverticillate to divaricate biverticillate smooth-walled conidiophores and quite slow-growing colonies, often with a brown reverse on some media. *P*. *ellipsoideosporum* differs from the other species by having longer and more divaricate metulae [65] and *P*. *shennongjianum* differs by having a higher proportion of conidiophores with three metulae [66]. The conidiophores of the latter two species are not vesiculate like most other species in the section.

Extrolite diagnosis of species like morphological recognition relies mostly on the predictable expression of the specific metabolites under standardized conditions. Each of these species has metabolites that can be used to identify the species.

Citreoviridin is a human lung tumor inhibitor [67] that may be of interest in finding new species that potentially produce other derivatives of citreoviridin in drug lead searches. Some derivatives of citreoviridin are citreomontanin [68], citreoviridinol and secocitreoviridin [69], neocitreoviridinol and epineocitreoviridinol [70], epiisocitreoviridinol [71], citreoviripyrone A and B [72], citreoviridin C and D [73] and herbarin A and B [74]. Citreoviridin production is not unique to section *Cinnamopurpurea*. We list all known citreoviridin producers in Table 1.

**Table 1:** Reported citreoviridin producers.

Reported producer	Actual identity	Reference
*Aspergillus alabamensis*	*Aspergillus alabamensis* (only IBT 29084)	[76]
*A*. *auroterreus*	*A*. *auroterreus*	[76]
*A*. *neoafricanus*	*A*. *neoafricanus*	[76]
*A niveus*	*A*. *neoniveus*?	[80, 81]
*A*. *pseudoterreus*	*A*. *pseudoterreus*	[79]
*A*. *terreus*	*A*. *terreus* (not all strains)	[73]
*Eupenicillium ochrosalmoneum*	*Penicillium ochrosalmoneum*	[16]
*Cladosporium herbarum*	Questionable record, should be confirmed	[74]
*Penicillium aurantiacobrunneum*	*P*. *aurantiacobrunneum*	[59]
*P*. *cairnsense*	*P*. *cairnsense*	[59]
*P*. *charlesii*	*P*. *colei*	[16, 17] corrected in this publication
*P*. *citreonigrum*	*P*. *citreonigrum*	[13]
*P*. *citreoviride*	*P*. *citreonigrum*	[3, 4, 82, 83]
*P*. *citrinum*	Probably *P*. *aurantiacobrunneum*, original strain not available	[84]
*P*. *colei*	*P*. *colei*	This report
*P*. *fellutanum*	Probably *P*. *citreonigrum*, strain not available	[85]
*P*. *gallaicum*	*P*. *gallaicum*	[59]
*P*. *godlewskii*	*P*. *godlewskii*	[59]
*P*. *isariiforme*	*P*. *isariiforme*	[79]
*P*. *manginii*	*P*. *manginii*	[78]
*P*. *miczynskii*	*P*. *miczynskii*	[78]
*P*. *monsseeratidens*	*P*. *monsserratidens*	This report
*P*. *neomiczynskii*	*P*. *neomiczynskii*	[59]
*P*. *pedemontanum*	*P*. *manginii*	[68]
*P*. *pulvillorum*	*P*. *manginii*	[4, 77, 78]
*P*. *quebecense*	*P*. *quebecense*	[59]
*P*. *smithii*	*P*. *smithii*	[78]
*P*. *vancouverense*	*P*. *vancouverense*	[59]
*P*. *toxicarium*	*P*. *toxicarium*	[86]

Two new species, *P*. *colei* and *P*. *monsserratidens* produce citreoviridin. The species are sibling and produce several other extrolites in common. They are separated because there is very strong statistical support for the concordance analysis that shows NRRL 13013 is neither conspecific with *P*. *monsserratidens* nor with the unstudied but related species *P*. *ellipsoideosporum*. Wicklow [75] using phenotypic species recognition thought it most likely that NRRL 13013 was a variant of *P*. *citreonigrum* in contrast to placement of the isolate in *P*. *charlesii* by Cole et al. [17]. Phylogenetic analysis shows that NRRL 13013 is not an isolate of *P*. *citreonigrum*, and also shows that it is distinct from its sibling species *P*. *monsserratidens* and *P*. *ellipsoideosporum*. Naming a species on the basis of a single isolate necessarily cannot describe all the variation (phenotypic or genetic) present in the species. NRRL 13013 produces citreoviridin and its identification has been subject to different interpretation as noted above. Here we resolve its identification by providing the new name *P*. *colei* to contain this isolate and establish that the most closely related previously described species is *Penicillium idahoense*, not *P*. *citreonigrum*. *Penicillium fluviserpens* isolates form two strongly supported subclades (Fig. 1), but were very similar phenotypically, have no outstanding characteristics or metabolites that would argue for distinct names and share some haplotypes, so we retain them in a single species. *Penicillium cvjetkovicii* isolates occur on a very strongly supported branch (Fig. 1) and share production of the extrolite AQ-840, but the individual isolates all produce additional different extrolites. NRRL 735 is an isolate obtained by Charles Thom from Biourge in 1924 as *P*. *griseo-roseum* and has been maintained in culture since. It differs from the other *P*. *cvjetkovicii* isolates by producing white, largely non-sporulating colonies. Peterson and Horn [27] noted the unusual growth of this isolate as others have [1, 4] and referred to it as *Penicillium* sp. until this study showed it to be *P*. *cvjetkovicii*. Other isolates of *P*. *cvjetkovicii* are quite consistent in appearance so the caution showed earlier [1, 4, 27] was justified. *Penicillium salmoniflumine* is represented by two isolates that produce the extrolite OTO1. NRRL 58001 also makes the unidentified metabolite endphe-1130. *Penicillium salmoniflumine* isolates are on a strongly supported branch and as a monophyletic group with distinguishing characters merits formal naming. The most problematic new species are *P*. *monsgalena* and *P*. *lemhiflumine*, each represented by a single isolate. *Penicillium monsgalena* NRRL 22302 (Fig. 1) could be grouped with *P*. *salmoniflumine* but does not share the common phenotype or genotype used to describe *P*. *salmoniflumine*.

Production of citreoviridin metabolites is not unique to the *P*. *idahoense* clade or even to the monoverticillate *Penicillium* species (Table 1). In *Penicillium* section *Citrina* the species *P*. *aurantioacobrunneum*, *P*. *citreonigrum*, *P*. *gallaicum*, *P*. *godlewskii*, *P*. *neomiczynskii*, *P*. *quebecense* and *P*. *vancouverense* [59] are producers. Citreoviridin is also produced by *Aspergillus terreus* [73], *A*. *aureoterreus*, *A*. *neoafricanus*, *A*. *pseudoterreus*, and *A*. *neoniveus* [76]. *Penicillium pulvillorum* was reported to produce citreoviridin [77], but the identification of the producer was later corrected to *P*. *manginii* [4, 78]. Reported citreoviridin production by *Cladosporium herbarum* [74] needs to be confirmed. Advanced chemical analytical techniques and genealogical concordance analysis of multilocus DNA sequence data allows confident assessment of the species that produce citreoviridin, and we speculate that the culture of *Cladosporium herbarum* was contaminated by a *Penicillium* or *Aspergillus* culture (see Table 1 for known producers) As noted above, citreoviridin contamination of foods is rarely reported and is not a major issue in food hygiene, however *P*. *ochrosalmoneum* can produce citreoviridin in maize, and *P*. *citreonigrum* can produce citreoviridin in rice, and has been claimed to induce Beri-beri disease [3,13,14–17, 19, 83–84, 86]. Of the species described here, *P*. *colei* was found on molded pecan fragments, and thus citreoviridin may occur in pecans. Generally it is of course not recommended to eat moldy nuts, and such nuts will probably be discarded by the producers during quality control of commercial nuts. *P*. *monsserratidens* has only been found in air samples, but we doubt it would give a mycotoxin problem via inhaling spores of this species, as it appears to be relatively infrequent in indoor air.

## Taxonomy

**Fig 4:**
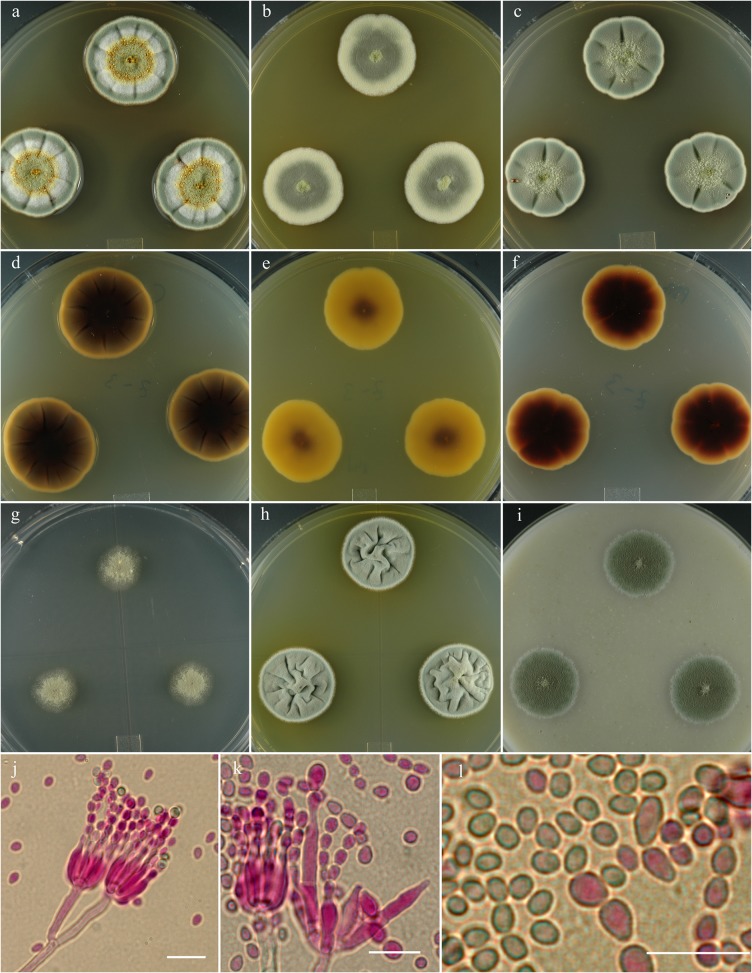
*Penicillium colei*. 14 day old colonies grown on: a. CYA, b. MEA, c. PDA. d. CYA reverse, e. MEA reverse, f. PDA reverse, g. CY20S, h. CYAS, i. OA. j. Stipes showing typical branching pattern, k. Penicillus with unusual multi-cell phialides, l. Conidia, small size is common, larger spores are also usually present. Bar = 10 μm.

***Penicillium colei*** S. W. Peterson, Ž. Jurjević & Frisvad, sp. nov. [urn:lsid:mycobank.org:names: 807368]; Fig. 4.

**Etymology:** Named in honor of Richard J. Cole, a noted mycotoxicologist and the discoverer of the type isolate of the species.

**Holotype:** Herb. BPI 881281.

**Diagnosis:** Distinguished from similar species by reverse color carob brown centrally to mouse gray marginally on CYA after 14 d.

**Barcode:** ITS = KF932958. Additional loci, beta tubulin KF932926, calmodulin KF932942, Mcm7 KF932975, RPB2 KF932996, Tsr1 KF933013.

**Colony morphology:** Colony diam, in mm, after 7d growth: CYA 5°C no growth; CYA 37°C no growth; culture incubated at 25°C: CYA 10–12; MEA 10; OA 9–10; CY20S 7–8; PDA 10–11; CYAS 10–11. Colony diam, in mm, after 14d growth: CYA 27–28; MEA 20; OA 20–21; CY20S 15–16; PDA 22–24; CYAS 22–23.

CYA yellow green (Vetiver green R47) in sporulating area with pale yellow margins, velutinous, moderately sulcate or wrinkled, mycelium white, occasionally yellowish, inconspicuous, exudate brownish orange, soluble pigments light-brown, no sclerotia, sporulation heavy, reverse dark brown nearly black centrally (carob brown R14), marginal area buff to nearly mouse gray (R51). MEA velutinous with central button 1–2 mm high, gray-blue-green (Castor gray R52), mycelium white to buff becoming pale yellow at the marginal, sporulation abundant, no exudate, no soluble pigment, no sclerotia, reverse brown-yellow centrally (ochraceous tawny R15) and pale yellow marginally (antimony yellow R15).

Conidiophores born from surface or aerial hyphae, stipes (15–) 30–150 (–300) × 3–4 μm, smooth-walled, with terminal irregular penicilli, irregularly apically swollen 2–5 (–8) μm diam, phialides in closely appressed verticils of 6–10, ampulliform to cylindrical (5–) 7–10 (–32) × 2–2.5 (–6) μm, with collula 1.5–3 μm long, conidia ellipsoidal to spherical 2.5–4 (–11) μm, smooth-walled, in well-defined columns.

**Specimens examined:** NRRL 13013 = IBT 29696: USA, Georgia, isolated from discarded pecan shell fragments, Sept. 1980, *R*. *J*. *Cole*, culture ex-type. IBT 21753: Denmark, Vibe, isolated from an air sample in a factory, *JC Frisvad*, culture.

**Note:** IBT 21753 produced the same extrolites as NRRL 13013.

**Metabolites:** AQ-840, citreomontanin, citreoviridin, OTO2, OTO5, pseurotin X, VYN, TUX, XOP.

*P*. *colei* is distinguished from similar species by reverse color carob brown centrally to mouse gray marginally on CYA after 14 d. The closest macro-morphological match is with *P*. *monsserratidens*. *P*. *colei* produces exudate on CYA and PDA in the center of colony while *P*. *monsserratidens* produces an abundance of exudate over the entire colony, mostly peripherally. Also *P*. *colei* does not produce exudate or soluble pigment on MEA while *P*. *monsserratidens* produces clear exudate and reddish yellow soluble pigment. Micro-morphological difference is that *P*. *colei* produces ellipsoidal to spherical 2.5–4 (–11) μm conidia, in contrast to *P*. *monsserratidens* that produces spherical to ellipsoidal conidia 2.5–3 (–7) μm.

**Fig 5:**
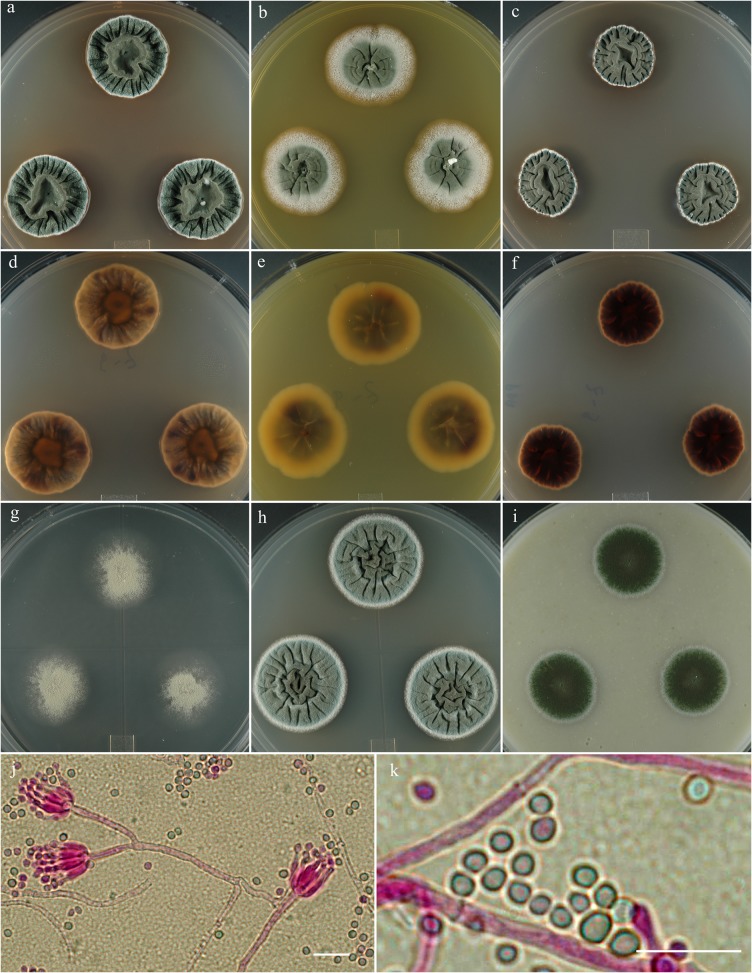
*Penicillium cvjetkovicii*. 14 day old colonies grown on: a. CYA, b. MEA, c. PDA. d. CYA reverse, e. MEA reverse, f. PDA reverse, g. CY20S, h. CYAS, i. OA. j. Details of penicillus branching pattern. k. Conidia in distinctly small and large sizes. Bar = 10 μm.

***Penicillium cvjetkovicii*** S. W. Peterson, Ž. Jurjević & Frisvad, sp. nov. [urn:lsid:mycobank.org:names:807369]; Fig. 5.

**Etymology:** Named for Croatian mycologist Bogdan Cvjetković.

**Holotype:** Herb. BPI 881283.

**Diagnosis:** Producing vinaceous to reddish-brown soluble pigments.

**Barcode:** ITS = KF932963. Additional loci, beta tubulin KF932931, calmodulin KF932948, Mcm7 KF932983, RPB2 KF933002, Tsr1 KF933021.

**Colony morphology:** Colony diam, in mm, after 7d growth: CYA 5°C no growth; CYA 37°C no growth; incubated at 25°C: CYA 8–15; MEA 7–14; OA 5–10; CY20S 5–11; PDA 8–16; CYAS 10–16. Colony diam, in mm, after 14d growth: CYA 17–28; MEA 12–25; OA 10–23; CY20S 11–26; PDA 12–28; CYAS 21–31.

CYA deep slate olive (R47) to light grayish olive (R47), velutinous, rising centrally 2–4 mm, sulcate, mycelium white, inconspicuous, sporulation abundant, no exudate, soluble pigment in vinaceous shades near Dresden brown (R15) through the entire Petri dish, no sclerotia, reverse vinaceous rufus (R14) to wood brown (R40). MEA dark bluish-gray-green (lily green R47), velutinous with central button, sporulation abundant, no exudate, faint red soluble pigment, no sclerotia, reverse brownish red to dark purplish brown-gray near cinnamon drab or Benzo brown (R46).

Conidiophores born from surface or aerial hyphae, stipes (5–) 15–60 (–150) × 1.8–2.2 μm, smooth-walled, apically swollen 4–5 (–10) μm, monoverticillate, phialides in verticils of (3–) 6–12 (–16), ampulliform (4–) 6–8 (–29) × 1.5–2.5 (–4.5) μm with short collula, conidia spherical to ellipsoidal (2–) 2.5–4 (–8) μm, with smooth to finely roughened walls, in irregular chains.

**Specimens examined:** NRRL 35841 = IBT 29714: USA, Northern California, isolated from an air sampler, Nov. 2007, by *Ž*. *Jurjević*, culture ex type: **metabolites**-AQ-840, OKF-745, PR-828, PR-878. NRRL 735 = IBT 29685: culture of unknown origin, ca 1923, contributed by *P*. *Biourge*: **metabolites**- AQ-840 (weak), OTO3, OTO6. NRRL 35903 = IBT 29689: USA, California, isolated from air sampler Sept. 2007, by *Ž*. *Jurjević* **metabolites**- AQ-840, PR-828, PR-878. NRRL 58240: USA, California, isolated from air sampler, Nov. 2008, *Ž*. *Jurjević*: **metabolites** *-*not tested.

**Notes:** One of us (ZJ) has commonly isolated this species from hospital environments in parts of the US. Despite this species’ isolation locales, it does not grow at body temperature. It is distinguished from similar species by producing faint red soluble pigment on MEA and CY20S to very strong, vinaceous to reddish-brown soluble pigments on CYA, PDA and CY5S. Macro-morphological appearance is unique. Growth on MEA with a central button, conidia dark bluish-gray-green, with abundant sporulation that looks almost powdery. At the margins it sporulates in ‘veins”. Mycelium at the margins subsurface or submerged into the media.

**Fig 6:**
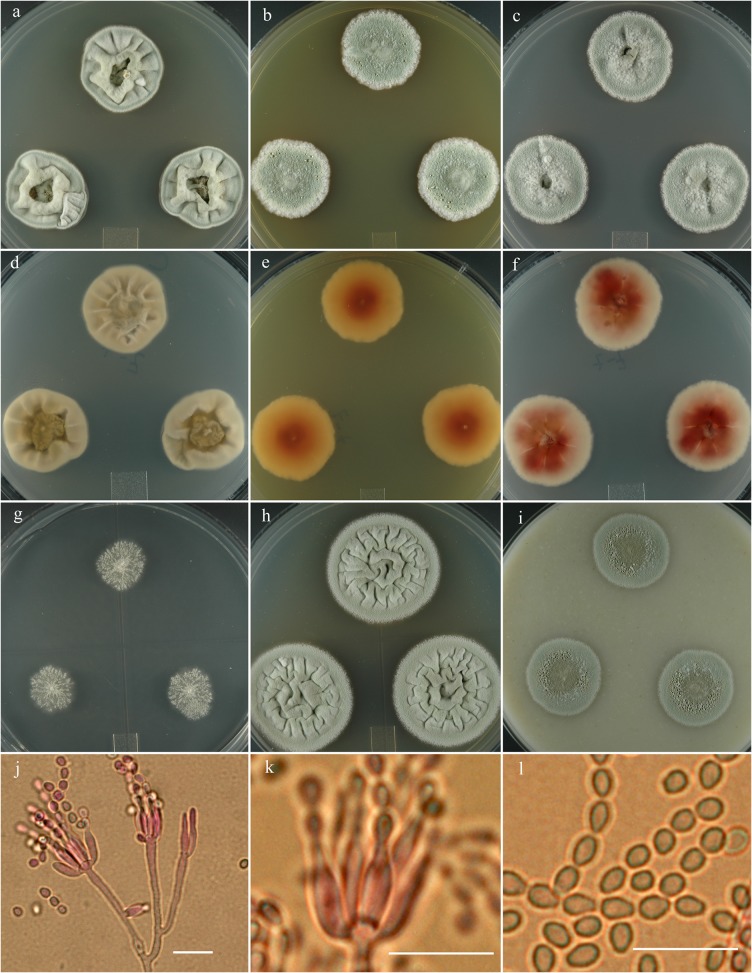
Penicillium fluviserpens. 14 day old colonies grown on: A. CYA, B. MEA, C. PDA. d. CYA reverse, e. MEA reverse, f. PDA reverse, g. CY20S, h. CYAS, i. OA. j. Terminal branching of the penicillus. k. Detail of ampulliform phialides and the bulbous end of the stipes. l. Conidia variable in size and shape. Bar = 10 μm.

**Penicillium fluviserpens** S. W. Peterson, Ž. Jurjević & Frisvad, sp. nov. [urn:lsid:mycobank.org:names: 807370]; Fig. 6.

**Etymology:** Named for the Snake River, Idaho USA.

**Holotype:** Herb. BPI 881284.

**Diagnosis:** Making no soluble pigment on MEA or CYA media.

**Barcode:** ITS = KF932961. Additional loci, beta tubulin KF932929, calmodulin KF932946, Mcm7 KF932981, RPB2 KF933000, Tsr1 KF933019.

**Colony morphology:** Colony diam, in mm, after 7 d growth: CYA 5°C no growth; CYA 37°C no growth; incubated at 25°C: CYA 10–12; MEA 8–11; OA 9–10; CY20S 5–9; PDA 10–12; CYAS 8–13. Colony diam, in mm, after 14 d growth: CYA 25–30; MEA 15–26; OA 17–23; CY20S 10–18; PDA 23–27; CYAS 21–32.

CYA pale gray-green (near tea green R47), velutinous, radially sulcate at margins and sulcate to wrinkled centrally, rising abruptly from the agar 3–5 mm, crateriform, mycelium white, clear exudate, abundant in some isolates, lacking in others, sporulation abundant, no soluble pigment, no sclerotia, reverse apricot orange (R15) or tawny or cinnamon orange (R14). MEA grayish greenish blue (celandine green R47), velutinous, low, lightly sulcate, centrally rising 1–2 mm, sporulation moderate, clear exudate sparse, light brownish soluble pigment in some isolates, brown globose sclerotia 200–550 μm diam present in NRRL 35844, reverse warm buff (R15) marginally, ochraceous orange (R15) centrally.

Conidiophores born from aerial hyphae, branching indeterminate, stipes (5–) 30–130 (–180) μm, smooth to finely roughened, monoverticillate apically swollen 5–6 (–10) μm diam, phialides in verticils commonly (6–) 8–12 (–16), ampulliform (5–) 6–8 (–32) × 2–3.5 μm, with long collula, conidia ellipsoidal to subspherical 2.5–3.5 (–7) μm, with walls smooth, in short irregular columns.

**Specimens examined**: NRRL 35838 = IBT 29686: USA, California, isolated from air sampler, Nov. 2007, by *Ž*. *Jurjević*, culture ex-type: **metabolites**- alk-752, AQ-840, OTO5, PR-828. NRRL 35844 = IBT 29687: USA, California, isolated from air sampler, Nov. 2007, *Ž*. *Jurjević*: **metabolites**- alk-752, AQ-840, ONX2, OTO5. NRRL 35848 = IBT 29683: USA, Pennsylvania, isolated from air sampler Feb. 2009, *Ž*. *Jurjević*: **metabolites**- alk-752, AQ-840, OTO4, OTO5. NRRL 58649: USA, Pennsylvania, isolated from air sampler Apr. 2009, *Ž*. *Jurjević*: **metabolites**-not tested.

**Notes:** *P*. *fluviserpens* has no soluble pigment on MEA or CYA media and occasionally produces brown globose sclerotia 200–550 μm diam on MEA after 14d.

**Fig 7:**
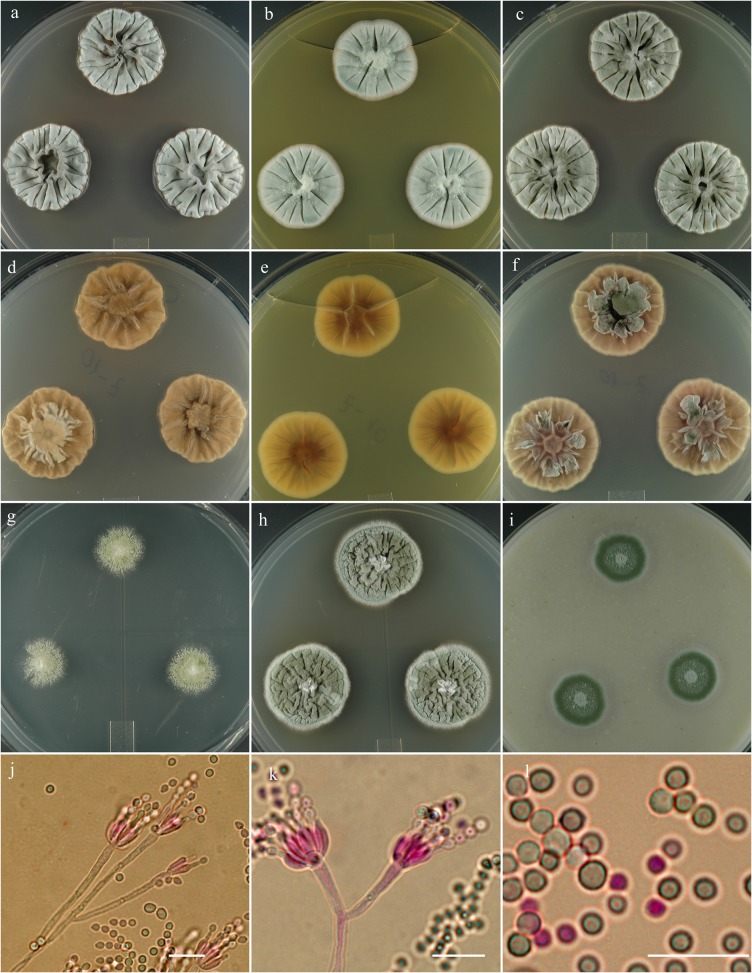
*Penicillium lemhiflumine*. 14 day old colonies grown on: a. CYA, b. MEA, c. PDA. d. CYA reverse, e. MEA reverse, f. PDA reverse, g. CY20S, h. CYAS, i. OA. j. Branching of penicillus. k. Phialides and non-bulbous subtending cell. l. Conidia in small and large sizes. Bar = 10 μm.

***Penicillium lemhiflumine*** S. W. Peterson, Ž. Jurjević & Frisvad, sp. nov. [urn:lsid:mycobank.org:names: 807371]; Fig. 7.

**Etymology:** Named for the Lemhi River in Idaho, a tributary to the Snake River.

**Holotype:** Herb. BPI 881287.

**Diagnosis:** Distinguished from similar species by formation of deeply sulcate colonies and sporulating area over the entire colony is gray green when grown on MEA.

**Barcode:** ITS = KF932964. Additional loci, beta tubulin KF932932, calmodulin KF932949, Mcm7 KF932984, RPB2 KF933003, Tsr1 KF933022.

**Colony morphology:** Colony diam, in mm, after 7 d growth: CYA 5°C no growth; CYA 37°C no growth; incubated at 25°C: CYA 12–13; MEA 10; OA 8–9; CY20S 7–8; PDA 13–14; CYAS 9–10. Colony diam, in mm, after 14 d growth: CYA 27–28; MEA 17; OA 17–18; CY20S 15–16; PDA 27–29; CYAS 26–28.

CYA composed of densely felted hyphae, deeply sulcate and wrinkled, rising centrally *ca* 5 mm, smooth surfaced, sporulating well, in color near Gnaphalium green (R 47), exudate absent, pinkish soluble pigment, reverse near Claret brown (R1) centrally and mars yellow (R3) near the margin. MEA sulcate, smooth, velutinous, sporulating well, in color near Celandine green (R47), exudate absent, faint pinkish soluble pigment, reverse color orange rufous (R2) to burnt sienna (R2).

Conidiophores arising from agar surface or from aerial hyphae, 20–250 μm × 2–3 μm, smooth-walled; penicilli monoverticillate, apically swollen 3–6 μm, with a terminal whorl of 5–10 phialides, phialides ampulliform, 5–7 μm × 1.5–2.0 μm with short collula, conidia spherical 3.0–3.5(–4.0) μm, smooth-walled, in irregular chains.

**Specimens examined:** NRRL 35843 = IBT 29684: USA, California, isolated from air sampler, Nov. 2007, by *Ž*. *Jurjević*, culture ex-type: **metabolites**-alk-752, AQ-840, OTO1, OTO4, OTO6.

**Notes:** *Penicillium lemhiflumine* is distinguished from similar species (*P*. *cvjetkovicii*, *P*. *salmoniflumine*, *P*. *monsgalena* and *P*. *idahoense*) that form sulcate colonies when growing on MEA. *Penicillium cvjetkovicii* growth on MEA occurs with a central button, conidia dark bluish-gray-green, with abundant sporulation that looks almost powdery. At the margins it sporulates in “veins”. Mycelium at the margins is subsurface or submerged in the media. In contrast the *P*. *lemhiflumine* sporulating area is gray green. *Penicillium lemhiflumine* grows twice as fast on CYA, MEA, OA, CY20S, PDA and CYAS media in contrast to *P*. *salmoniflumine* which has good growth only on CYAS. *Penicillium monsgalena* has a gray-green conidial area at the center of the colony, while *P*. *lemhiflumine* sporulates heavy over the entire colony and radial sulcation is very pronounced. *Penicillium idahoense* on CYA produces abundant brown to dark brown sclerotia, 200–300μm diameter, clear exudate, abundant; no soluble pigments; reverse purple to dark purplish brown. On MEA it produces brown sclerotia, 100–300 μm in diameter. In contrast, *P*. *lemhiflumine* produces pinkish soluble pigment on CYA and does not produce sclerotia on CYA or MEA.

**Fig 8:**
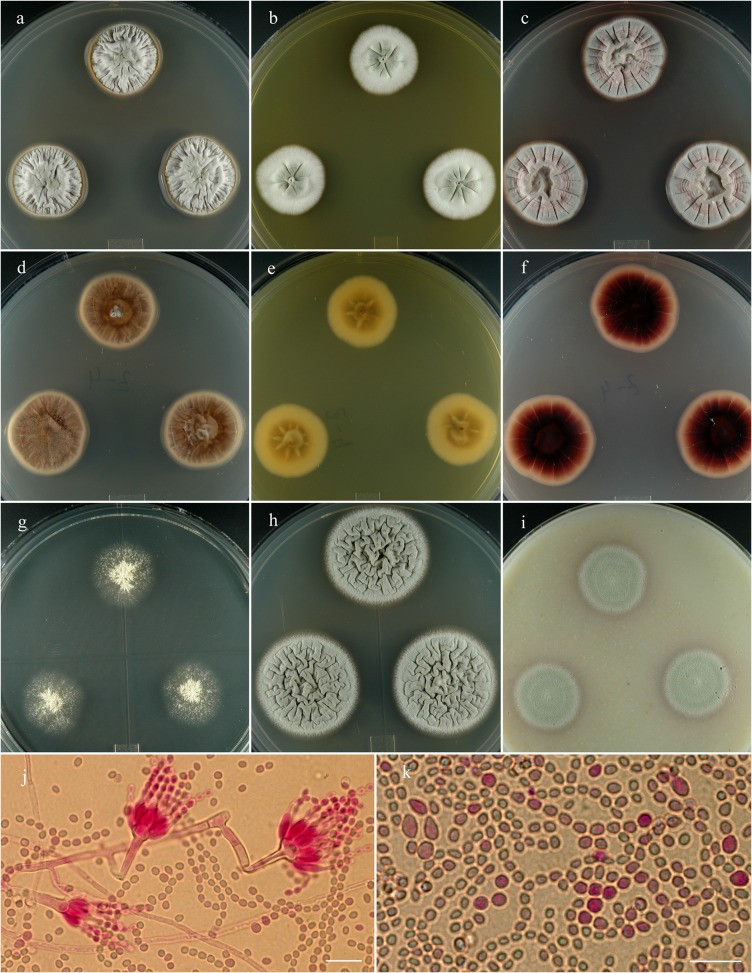
*Penicillium monsgalena*. 14 day old colonies grown on: a. CYA, b. MEA, c. PDA. d. CYA reverse, e. MEA reverse, f. PDA reverse, g. CY20S, h. CYAS, i. OA. j. Penicilli with a bulbous subtending cell, on short lateral branches from a primary filament. k. Mass of conidia showing distinctly different sizes. Bar = 10 μm.

**Penicillium monsgalena** S. W. Peterson, Ž. Jurjević & Frisvad, sp. nov. [urn:lsid:mycobank.org:names: 807372]; Fig. 8.

**Etymology:** Named for Mt. Galena, the headwater area of the Salmon River, Idaho, USA.

**Holotype:** Herb. BPI 881282.

**Diagnosis:** Distinguished from similar species by distinctly gray-green conidial area at the center of the colony and fleshy reverse color of colonies grown on CYA

**Barcode:** ITS = KF932959. Additional loci, beta tubulin KF932927, calmodulin KF932943, Mcm7 KF932977, RPB2 KF932997, Tsr1 KF933015.

**Colony morphology:** Colony diam, in mm, after 7 d growth: CYA 5°C no growth; CYA 37°C no growth; incubated at 25°C: CYA 10–12; MEA 10; OA 10; CY20S 7–9; PDA 11–12; CYAS 12–13. Colony diam, in mm, after 14 d growth: CYA 24–25; MEA 20; OA 20; CY20S 20; PDA 25–27; CYAS 30–31.

CYA radially sulcate, moderately raised with central depression, consisting of a dense felt, sporulation moderate, gray-green near Court gray (R47), no exudate, no soluble pigment, reverse castor gray (R62) mottled with pale yellow drab (R2) or tawny color. MEA velutinous, radially light to moderate deep sulcate, slightly raised centrally, consisting of a field of densely packed conidiophores, sporulation copious, gray-green (Court gray R47) or light celandine green (R47), no exudate, no soluble pigments, reverse pale orange-red to mikado orange (R2).

Conidiophores arising from aerial or basal hyphae 40–100 × 2.5–4 μm, smooth-walled, irregularly branched, monoverticillate, occasionally furcate, apically swollen 5–7 μm diam, with whorl of 6–10 divergent phialides 3–3.5 × 8–10 μm, conidia ellipsoidal to subglobose (2.5–) 3–4 (–5) μm diam, smooth walled.

**Specimens examined:** NRRL 22302 = IBT 29713: South Africa, isolated from corn meal, prior to 1965, culture ex-type: **metabolites**-AQ-840, OTO4, PR-828, PR-878.

**Notes:** Distinguished from similar species by distinctly gray-green conidial area and fleshy reverse color of colonies grown on CYA. Also produces purplish red soluble pigment on OA. *Penicillium monsserratidens*, and *P*. *salmoniflumine* produce reddish soluble pigments on OA as well. *Penicillium monsgalena* grows twice as fast as *P*. *salmoniflumine*, and *P*. *monsgalena* has velutinous growth with light to moderate deep sulcation on MEA while *P*. *salmoniflumine* is deeply sulcate to wrinkled on MEA. *P*. *monsgalena* produces larger conidia in average; ellipsoidal to subglobose (2.5–) 3–4 (–5) μm diam, in contrast to *P*. *monsserratidens* with conidia spherical to ellipsoidal 2.5–3 (–7) μm and *P*. *salmoniflumine* conidia ellipsoidal to spherical (2–) 2.5–3.5 (–6) μm.

**Fig 9:**
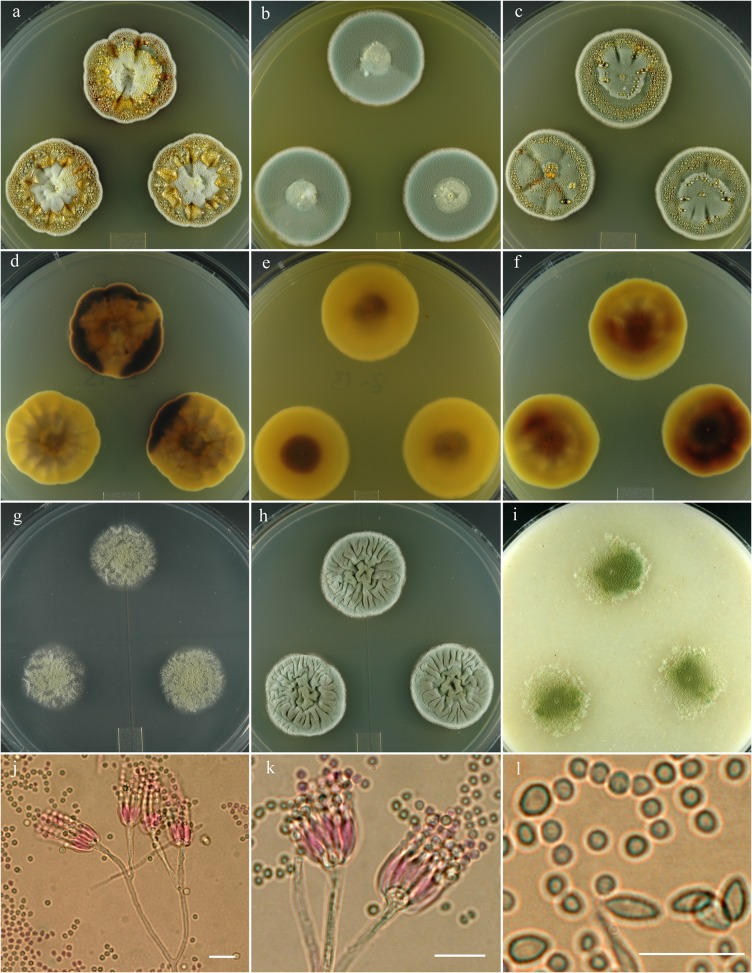
*Penicillium monsserratidens*. 14 day old colonies grown on: a. CYA, b. MEA, c. PDA. d. CYA reverse, e. MEA reverse, f. PDA reverse, g. CY20S, h. CYAS, i. OA. j. Branching pattern of penicillus. k. Phialides and bulbous subtending cell. l. Distinctly different size and shapes of conidia, the smaller subglobose-globose cells are most common. Bar = 10 μm.

**Penicillium monsserratidens** S. W. Peterson, Ž. Jurjević & Frisvad, sp. nov. [urn:lsid:mycobank.org:names: 807373]; Fig. 9.

**Etymology:** Named for the Sawtooth Mountain range of central Idaho.

**Holotype:** Herb. BPI 881285.

**Diagnosis:** Distinguished from similar species by copious clear to orange exudate, very dark vinaceous brown reverse and reddish brown soluble pigment produced by colonies grown on CYA.

**Barcode:** ITS = KF932962. Additional loci, beta tubulin KF932930, calmodulin KF932947, Mcm7 KF932982, RPB2 KF933001, Tsr1 KF933020.

**Colony morphology:** Colony diam, in mm, after 7 d growth: CYA 5°C no growth; CYA 37°C no growth; incubated at 25°C: CYA 10–14; MEA 9–13; OA 8–10; CY20S 9–10; PDA 9–14; CYAS 10–13. Colony diam, in mm, after 14 d growth: CYA 20–29; MEA 18–26; OA 13–24; CY20S 15–23; PDA 19–27; CYAS 25–30.

CYA dull green (Vetiver green R47), velutinous, sulcate to wrinkled centrally, crateriform, margins sunken 0.5–1 mm, mycelium white to yellowish-orange, sporulation abundant, exudate yellowish, brown to dark red brown, often abundant, soluble pigment yellowish brown to reddish brown, no sclerotia, reverse vinaceous-rufous (R14) to purplish brown (dusky purplish gray R53) nearly black. MEA blue-green (tea green R47), velutinous, plain, low, rising centrally 1–2 mm, sporulation abundant, exudate clear and sparse in some isolates (NRRL 62003), no sclerotia, soluble pigment reddish yellow-brown, reverse usually red-brown (burnt Sienna R2) centrally, tawny or yellow marginally.

Conidiophores born from surface or aerial hyphae, stipes branching indeterminate, (20–) 35–150 (–280) × 3–4 μm, with smooth walls, monoverticillate, apically swollen (2–) 4–6 (–8) μm diam, bearing whorls of (6–) 8–12 (–16) phialides, phialides ampulliform (5–) 6–8 (–28) × 1.5–2.5 (–3.5) μm with short collula, conidia spherical to ellipsoidal 2.5–3 (–7) μm with smooth walls, born in irregular chains.

**Specimens examined:** NRRL 35884 = IBT 29695: USA, California, isolated from air sampler, Nov. 2007, *Ž*. *Jurjević*, culture ex-type: **metabolites**-AQ-840, citreomontanin, citreoviridin, OTO2, OTO5, pseurotin X, VYN, XOP. NRRL 35840 = IBT 29694: USA, California, isolated from air sampler, Nov. 2007, *Ž*. *Jurjević*: **metabolites**-AQ-840, citreomontanin, citreoviridin, OTO2, pseurotin X, TUX, XOP. NRRL 62003: USA, Idaho, isolated from air sampler, Feb. 2010, *Ž*. *Jurjević*: **metabolites**-not tested.

**Notes:** Distinguished from similar species by copious clear to orange exudate, very dark vinaceous brown reverse and reddish brown soluble pigment produced by colonies grown on CYA. The closest species with similar macro-morphological appearance is *P*. *colei* but *P*. *colei* produces exudate on CYA and PDA in the center of the colony while *P*. *monsserratidens* produces an abundance of exudate over the entire colony, mostly peripherally. Also *P*. *colei* does not produce exudate or soluble pigment on MEA while *P*. *monsserratidens* produces clear exudate and reddish yellow soluble pigment. In micro-morphology *P*. *cvjetkovicii* produces ellipsoidal to spherical conidia 2.5–4 (–11) μm, while *P*. *monsserratidens* conidia are spherical to ellipsoidal 2.5–3 (–7) μm.

**Fig 10:**
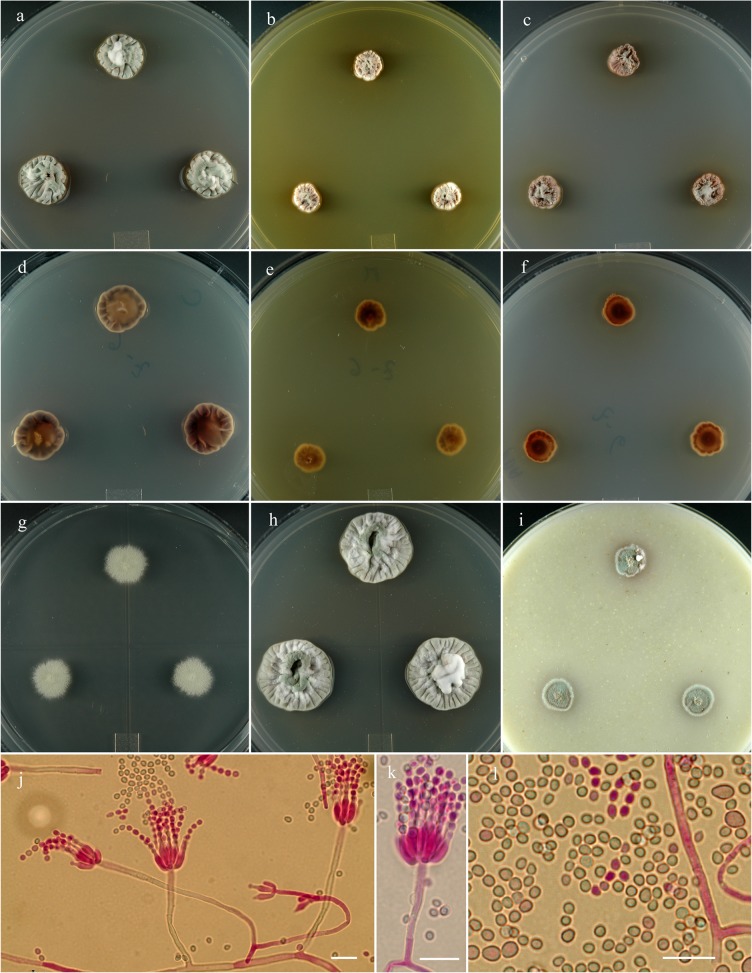
*Penicillium salmoniflumine*. 14 day old colonies grown on: a. CYA, b. MEA, c. PDA. d. CYA reverse, e. MEA reverse, f. PDA reverse, g. CY20S, h. CYAS, i. OA. j. Branching pattern of penicillus. k. Detail of phialides and stipes. l. Conidia in two size classes. Bar = 10 μm.

**Penicillium salmoniflumine** S. W. Peterson, Ž. Jurjević & Frisvad, sp. nov. [urn:lsid:mycobank.org:names: 807374]; Fig. 10.

**Etymology:** Named for the Salmon River in Idaho, USA.

**Holotype:** Herb. BPI 881286.

**Diagnosis:** Distinguished from similar species by small colonies with bright red soluble pigments, good growth only on CYAS.

**Barcode:** ITS = KF932960. Additional loci, beta tubulin KF932928, calmodulin KF932945, Mcm7 KF932980, RPB2 KF932999, Tsr1 KF933018.

**Colony morphology:** Colony diam, in mm, after 7 d growth: CYA 5°C no growth; CYA 37°C no growth; incubated at 25°C: CYA 5–7; MEA 4–7; OA 4–5; CY20S 5–8; PDA 5–6; CYAS 10. Colony diam, in mm, after 14 d growth: CYA 11–16; MEA 7–10; OA 10; CY20S 13–15; PDA 9–10; CYAS 21–25.

CYA composed of a thick mycelial mat, light cinnamon drab (R46), rising abruptly ca 2 mm above the agar, moderately to deeply sulcate and wrinkled, mycelium white and inconspicuous, sporulation good, making Gnaphalium green color (R47), no exudate, soluble pigment vinaceous, no sclerotia, reverse initially mouse gray to deep mouse gray (R51) becoming deep red-brown in age. MEA light cinnamon drab (R46) mycelial mat, rising ca 1–2 mm above agar, sulcate to wrinkled, sporulation abundant and in Gnaphalium green (R47), no exudate, soluble pigment brownish inconspicuous, no sclerotia, reverse vinaceous drab to deep vinaceous drab (R45).

Conidiophores arise from surface or aerial hyphae, stipes 15–250 μm, smooth to finely roughened walls, monoverticillate, apically swollen 3–6 (–9) μm diam, phialides in verticils of (4–) 6–12 (–16), rarely producing monophialides, phialides acerose to ampulliform (5–) 6–8 (–27) × 1.5–2.5 (–6) μm, with short collula, conidia ellipsoidal to spherical (2–) 2.5–3.5 (–6) μm, with smooth to finely roughened walls, in loose to well defined columns.

**Specimens examined:** NRRL 35837 = IBT 29673: USA, California, isolated from an air sampler, Nov. 2007, *Ž*. *Jurjević*, culture ex-type: **metabolites**-AQ-840, OTO1, OTO4. NRRL 58001 = IBT 29688: USA, California, isolated from an air sampler, Mar. 2008, *Ž*. *Jurjević*: **metabolites**-AQ-840, OTO1, OTO4, endphe-1130.

**Fig 11:**
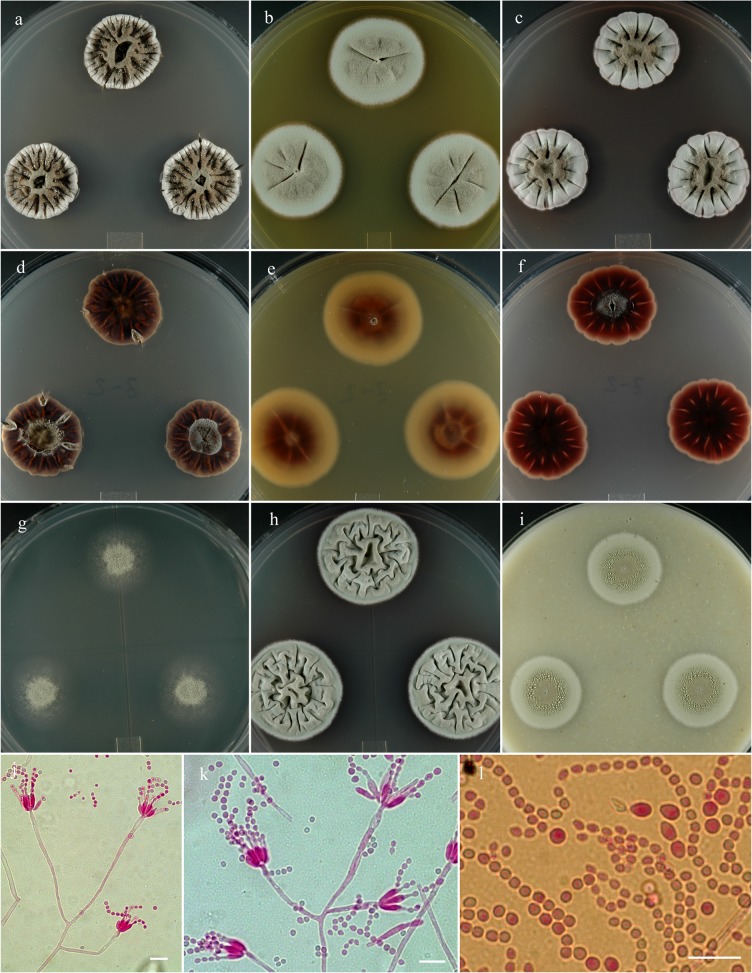
*Penicillium idahoense*. 14 day old colonies grown on: a. CYA, b. MEA, c. PDA. d. CYA reverse, e. MEA reverse, f. PDA reverse, g. CY20S, h. CYAS, i. OA. j. Branching of penicillus. k. Detailed view of phialides and stipes. l. Conidia displaying two size ranges. Bar = 10 μm.

An array of photographs and microphotographs of *Penicillium idahoense* is included here for comparative purposes (Fig. 11).

## Supporting Information

S1 Datafile Beta tubulin alignment as a Mega data file. (TXT)

S2 Datafile Calmodulin alignment as a Mega data file. (TXT)

S3 Datafile ITS alignment as a Mega data file. (TXT)

S4 Datafile Mcm7 alignment as a Mega data file. (TXT)

S5 Datafile RPB2 alignment as a Mega data file. (TXT)

S6 Datafile Tsr1 alignment as a Mega data file. (TXT)

S1 Fig Single locus Trees. Single locus trees calculated from the listed locus and mega likelihood method, with bootstrap values shown by: green branches 90–100%, yellow 80–89% and red 70–79%.
(PDF)

S1 Table Provenance of isolates. (DOCX)

S2 Table GenBank Numbers. Species, isolates and associated GenBank numbers.
(DOCX)
