# Supplementary material for: Expanding the Species and Chemical Diversity of Penicillium Section Cinnamopurpurea
Source: PLoS One. 2015 Apr 8;10(4):e0121987. doi: 10.1371/journal.pone.0121987 (PMC4390383; doi:10.1371/journal.pone.0121987)
Supplement: S1 Fig — Single locus trees calculated from the listed locus and mega likelihood method, with bootstrap values shown by: green branches 90–100%, yellow 80–89% and red 70–79%. (PDF) [file pone.0121987.s007.pdf]

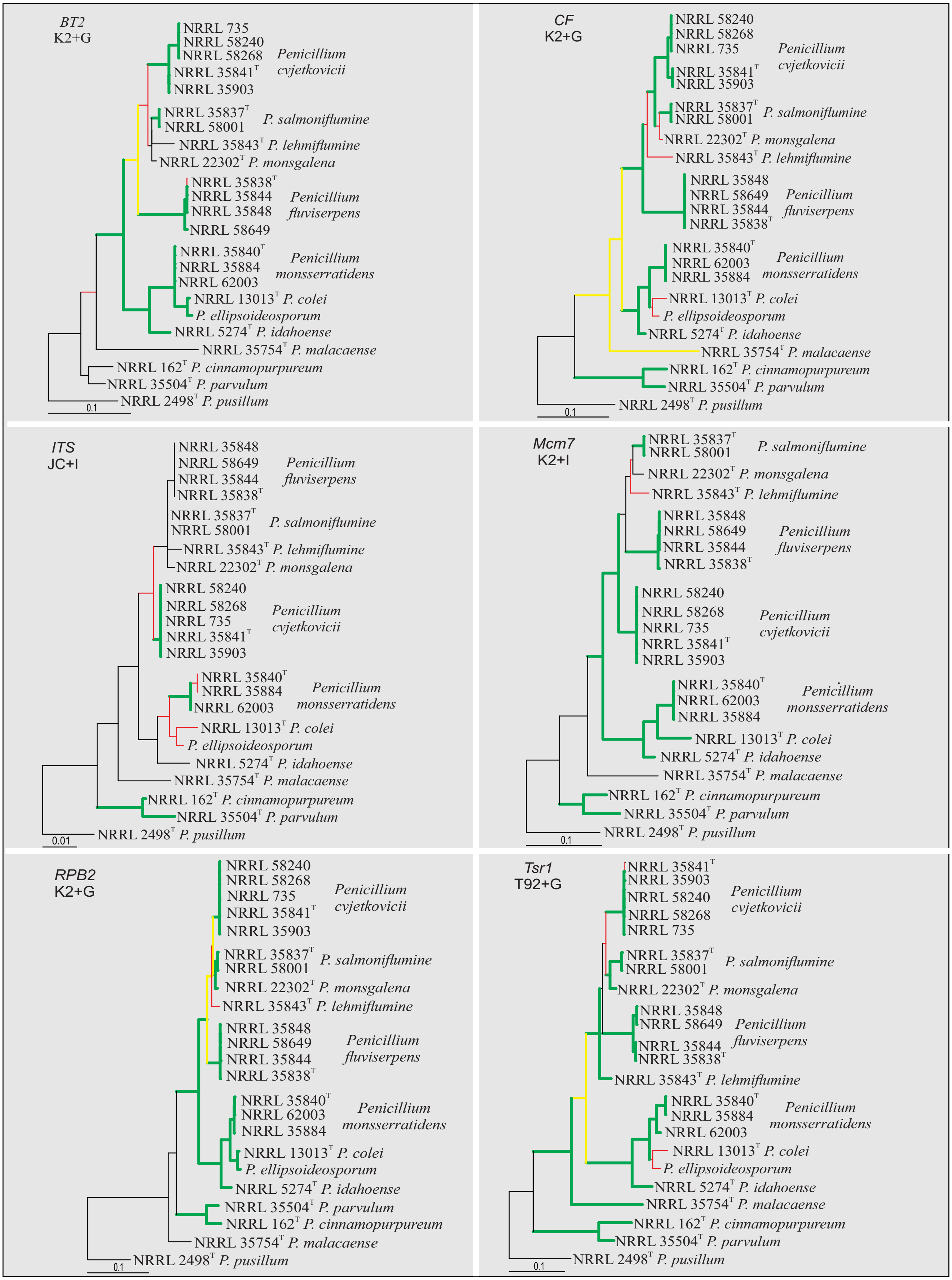

Green lines=90-100% bootstrap; yellow lines=80-89% bootstrap; redlines=70-79% bootstrap values, black lines represent bootstrap values lower than 70%. Locus and analytical model in each box.
