# Supplementary material for: Expanding the Species and Chemical Diversity of Penicillium Section Cinnamopurpurea
Source: PLoS One. 2015 Apr 8;10(4):e0121987. doi: 10.1371/journal.pone.0121987 (PMC4390383; doi:10.1371/journal.pone.0121987)
Supplement: S1 Table — (DOCX) [file pone.0121987.s008.docx]

S1 Table. Provenance of isolates used in this study.

| NRRL Accession No. | Isolation data |
| --- | --- |
| *Penicillium cinnamopurpureum* | |
| 162 | Japan, isol ex milled rice, 1960, *S. Udagawa*, ex type |
| 3118 | USA, Kansas, isol ex wheat, 1964, *R. Graves*. |
| 3226 | India, 1966, ex type of teleomorph. |
| 22293 | Australia, isol ex toxic swine feed, 1993. |
| 35500 | USA, Geogia, isol ex peanut filed soil, 2004, *B. W. Horn*. |
| 35501 | USA, Geogia, isol ex peanut filed soil, 2004, *B. W. Horn*. |
| 35502 | USA, Geogia, isol ex peanut filed soil, 2004, *B. W. Horn*. |
| *Penicillium colei* | |
| 13013 | USA, Georgia, isol ex discarded pecan shells, 1981, *RJ Cole*, ex type |
| *Penicillium cvjetkovicii* | |
| 735 | Unknown, contributed by *P Biourge* ca 1923. |
| 35841 | USA, California, isol ex air, 2007, *Z Jurjevic*. |
| 35903 | USA, California, isol ex air, 2007, *Z Jurjevic*. |
| 58240 | USA, California, isol ex air, 2008, *Z Jurjevic*. |
| *Penicillium fluviserpens* | |
| 35838 | USA, California, isol ex air, 2007, *Z Jurjevic*. |
| 35844 | USA, California, isol ex air, 2007, *Z Jurjevic*. |
| 35848 | USA, California, isol ex air, 2007, *Z Jurjevic*. |
| 58649 | USA, Pennsylvania, isol ex air, 2009, *Z Jurjevic*. |
| *Penicillium idahoense* | |
| 5274 | USA, Idaho, Latah county, isol ex soil, 1966, *JW Paden*, ex type. |
| *Penicillium lemhiflumine* | |
| 35843 | USA, California, isol ex air, 2007, *Z Jurjevic*. |
| *Penicillium malacaense* | |
| 35754 | Spain, Madrid, isol ex air, 1979, *C Ramirez*, ex type. |
| *Penicillium monsgalena* | |
| 22302 | South Africa, isol ex corn meal, ca 1965. |
| *Penicillium monsserratidens* MB | |
| 35840 | USA, California, isol ex air, 2007, *Z Jurjevic*. |
| 35884 | USA, California, isol ex air, 2007, *Z Jurjevic*. |
| 62003 | USA, Idaho, isol ex air, 2010, *Z Jurjevic*. |
| *Penicillium parvulum* | |
| 35504 | USA, Georgia, isol ex peanut seed, 2004, *B. W. Horn*. |
| *Penicillium pusillum* | |
| 2498 | UK, isol ex dried peas, 1947, *G. Smith*, ex type. |
| *Penicillium salmoniflumine* MB | |
| 35837 | USA, California, isol ex air, 2007, *Z Jurjevic*. |
| 58001 | USA, California, isol ex air, 2008, *Z Jurjevic*. |
