# Supplementary material for: Expanding the Species and Chemical Diversity of Penicillium Section Cinnamopurpurea
Source: PLoS One. 2015 Apr 8;10(4):e0121987. doi: 10.1371/journal.pone.0121987 (PMC4390383; doi:10.1371/journal.pone.0121987)
Supplement: S2 Table — Species, isolates and associated GenBank numbers. (DOCX) [file pone.0121987.s009.docx]

S2 Table. Isolates, loci and GenBank accession numbers for the DNA sequence data used in calculating the phylogenetic trees. Missing data is depicted with a dash.

| Species | NRRL no. | *BT2* | *Calmodulin* | *ITS* | *Mcm7* | *RPB2* | *Tsr1* |
| --- | --- | --- | --- | --- | --- | --- | --- |
| *Penicillium cinnamopurpureum* | 162 | EF626948 | EF626949 | EF626950 | KF932976 | EF626952 | KF933014 |
| *Penicillium colei* | 13013 | KF932926 | KF932942 | KF932958 | KF932975 | KF932996 | KF933013 |
| *Penicillium cvjetkovicii* | 735 | EF506221 | EF506233 | AF033413 | KF932994 | KF933011 | KF933031 |
|  | 35841 | KF932931 | KF932948 | KF932963 | KF932983 | KF933002 | KF933021 |
|  | 35903 | KF932936 | KF932953 | KF932968 | KF932988 | KF933007 | KF933025 |
|  | 58240 | KF932937 | KF932955 | KF932970 | KF932991 | — | KF933028 |
|  | 58268 | KF932938 | KF932956 | KF932971 | — | — | — |
| *Penicillium fluviserpens* | 35838 | KF932929 | KF932946 | KF932961 | KF932981 | KF933000 | KF933019 |
|  | 35844 | KF932933 | KF932950 | KF932965 | KF932985 | KF933004 | KF933023 |
|  | 35848 | KF932934 | KF932951 | KF932966 | KF932986 | KF933005 | KF933024 |
|  | 58649 | KF932939 | — | KF932972 | KF932992 | KF933009 | KF933029 |
| *Penicillium idahoense* | 5274 | EF626953 | EF626954 | EF626955 | KF932989 | EF626956 | KF933026 |
| *Penicillium lehmiflumine* | 35843 | KF932932 | KF932949 | KF932964 | KF932984 | KF933003 | KF933022 |
| *Penicillium malacaense* | 35754 | EU427268 | KF932944 | EU427300 | KF932979 | EU427261 | KF933017 |
| *Penicillium monsgalena* | 22302 | KF932927 | KF932943 | KF932959 | KF932977 | KF932997 | KF933015 |
| *Penicillium monsserratidens* | 35840 | KF932930 | KF932947 | KF932962 | KF932982 | KF933001 | KF933020 |
|  | 35884 | KF932935 | KF932952 | KF932967 | KF932987 | KF933006 | — |
|  | 62003 | KF932940 | KF932957 | KF932973 | KF932993 | KF933010 | KF933030 |
| *Penicillium salmoniflumine* | 35837 | KF932928 | KF932945 | KF932960 | KF932980 | KF932999 | KF933018 |
|  | 58001 | — | KF932954 | KF932969 | KF932990 | KF933008 | KF933027 |
| *Penicillium parvulum* | 35504 | EF506218 | EF506225 | EF422845 | KF932978 | — | KF933016 |
| *Penicillium pusillum* | 2498 | KF932925 | KF932941 | EF626951 | KF932974 | KF932995 | KF933012 |
| *Penicillium ellipsoideosporum* |  | JQ965104 | AY678559 | JX012224 | - | JN121427 | JN121734 |
